# Juggling two pandemics: The simultaneous necessity and difficulty of practising lifestyle medicine during the COVID‐19 era

**DOI:** 10.1002/lim2.68

**Published:** 2022-08-03

**Authors:** Alexandra Shipley, Ellen Fallows

**Affiliations:** ^1^ Medical Sciences Division Oxford University Oxford UK; ^2^ British Society of Lifestyle Medicine Northants UK

**Keywords:** exercise, lifestyle medicine, nutrition, relationships, stress

## Abstract

Since early 2020, COVID‐19 has dominated headlines, claimed millions of lives, crippled global economies, overwhelmed health services, attracted multi‐disciplinary scientific attention and transformed our daily lives. Unsurprisingly, the Lifestyle Medicine field has not been immune to the pandemic's wide‐reaching influence. Although COVID‐19 highlighted the necessity of maintaining healthy behaviours, the associated lockdowns and social distancing measures challenged our ability to do so. Attempts to mitigate the spread of COVID‐19 may, therefore, have exacerbated the obesity pandemic and other diseases associated with unhealthy lifestyle habits. One hopes this devastating virus provides the impetus for policymakers, clinicians and patients to collaborate in tackling the diseases of modern life. This commentary explores how lifestyle‐correlated conditions (which are closely intertwined with socioeconomic factors) rendered much of the UK population vulnerable to COVID‐19 infection, morbidity and mortality. Subsequently, we consider the impact of lockdown measures on the accessibility of healthy living, focussing on eating behaviours, physical activity, relationships, sleep and substance abuse, as well as the social demographics particularly affected. Approaching the aftermath of this vicious cycle with optimism, we discuss why the post‐Covid era presents a unique opportunity for Lifestyle Medicine, as an evidence‐based approach to supporting patients to adopt and sustain healthy behaviours.

## 1 INTRODUCTION

Early data indicate that smoking, physical inactivity and obesity account for 51% of the population‐attributable fraction of coronavirus disease 2019 (COVID‐19) hospitalisations.^1^ Meanwhile, lockdown measures (implemented to curb severe acute respiratory syndrome coronavirus 2 (SARS‐CoV‐2) transmission) challenged the ability of UK residents to maintain healthy lifestyles. Focussing on data from the UK, this commentary explores the bidirectional relationship between unhealthy behaviours and the impact of COVID‐19. Subsequently, we explore how Lifestyle Medicine can define its role and establish its niche within the rapidly evolving healthcare landscape emerging from the height of the COVID‐19 pandemic.

## 2 THE INFLUENCE OF LIFESTYLE BEHAVIOURS ON COVID‐19 INFECTION AND MORBIDITY

Mounting a targeted response to any disease outbreak requires epidemiological identification of vulnerable demographics; a prospective cohort study investigating 387,109 UK adults revealed that obesity and certain lifestyle factors (smoking, physical inactivity and excess alcohol) augment the risk of both infection and hospitalisation.^1^ In particular, obesity increases the risk of critical illness from COVID‐19 by 97%,^2^ which could be explained by constitutive up‐regulation of pro‐inflammatory factors, compromised leukocyte function (due to lipid deposition in lymphoid tissue) and over‐expression of Angiotensin Converting Enzyme‐2 (ACE‐2) (the transmembrane enzyme exploited by SARS‐CoV‐2 for cell penetration).^3^ Indeed, comparing the UK with international populations reveals a linear correlation between prevalence of obesity in a country and COVID‐19 mortality.^4^

In terms of the lifestyle factors contributing to obesity, a Western diet has been shown to increase SARS‐CoV‐2 morbidity in a hamster model.^5^ Although animal models offer limited translatability to humans, this finding is supported by the fact that plant‐based and pescatarian diets confer a lower risk of complications.^6^ Equally, moderate‐intensity exercise supports healthy immune function^7^ (by boosting leukocyte function and up‐regulating anti‐inflammatory cytokines) and decelerates immune senescence with ageing: a particularly vulnerable demographic.^8^

Considering other pillars of Lifestyle Medicine, sleep deprivation and cortisol (released upon stress) both supress immune function. A systematic review associated <7 h sleep/night with a 31% increased risk of upper respiratory tract infections (with the obvious caveat that activities limiting sleep duration may have contributed).^9^ Meanwhile, a prospective cohort study correlated psychological distress during the early pandemic with subsequent infection risk and symptom severity.^10^ Smokers are more likely to develop symptomatic COVID‐19,^11^ while alcohol consumption increases the risk of infection physiologically (by up‐regulating ACE‐2 expression)^12^ and behaviourally (by reducing compliance with social‐distancing).^13^ This highlights the urgent demand for Lifestyle Medicine, prompting us to consider how the field might harness society's renewed interest in health to establish itself as a cornerstone of modern culture.

## 3 THE IMPACT OF COVID‐19 ON HEALTHY LIVING

In a Public Health England survey, 41% respondents reported weight‐gain; averaging 4.1 kg over the 16 months after national lockdown was introudced.^14^ Whilst participants commonly attributed this to snacking and comfort eating, a more nuanced range of psychological and behavioural factors may be implicated. For example, 49% reported increased binge‐eating, which may reflect loss of routine, accessibility of food at home or triggers such as stress (aroused by health concerns, social isolation, carer responsibilities and financial uncertainty).^15^ In fact, within 2 months of national lockdown, 48% felt more anxious and depressed,^16^ which may have affected eating behaviours, as well as emotional wellbeing, substance abuse, sleep and exercise.

Indeed, lockdown brought a 20% decrease in the number of days, where adults exercised >30 min, likely due to restrictions on team/racket sports, closure of gyms/pools, reduced commuting and prolonged childcare demands.^17^ Furthermore, the UK is the only European country in which alcohol consumption increased,^18^ with the 25% increase in sales likely contributing to the 20% increase in alcohol‐specific deaths in 2020.^19^ Similarly, there was a surge in smoking, with over 650,000 18–34‐year‐olds adopting the habit.^20^ Social media use (considered another addictive harmful behaviour) soared 72%,^21^ potentially in an attempt to alleviate the ‘loneliness’,^22^ and ‘disturbed sleep,^23^ induced by national lockdown in 36% and 50% of survey respondents, respectively.

Vicious cycling of these concomitant pandemics is alarming; diseases associated with lifestyle increase COVID‐19 vulnerability, driving stricter and lengthier lockdowns (to avoid overburdening the already‐stretched health services). In turn, these measures further challenge our ability to maintain physically/emotionally healthy lifestyles, prompting behaviours that compromise immunity.

## 4 COVID‐19 AS AN AMPLIFIER OF SOCIAL INEQUALITIES

The impact of COVID‐19 on lifestyle behaviours has not been uniform across all demographics, exacerbating health inequalities already endemic within British society. Exercise participation and fruit/vegetable consumption were disproportionately reduced amongst ethnic minorities and individuals of a lower education attainment.^24^ The fact that groups at greatest risk of COVID‐19 morbidity experienced the greatest barriers to healthy living during lockdown epitomises the vicious cycle. For example, survey participants with a greater body mass index (BMI) were more likely to report additional challenges in accessing a nutritious diet, as well as reduced social support and self‐control around food during lockdown.^25^ Furthermore, it may be particularly challenging to overcome unhealthy habits entrained during a COVID‐disrupted childhood. In 2021, The National Child Measurement Programme recorded the greatest yearly increase in overweight/obesity levels amongst 10–11‐year‐olds to date (now 40.9%), with children from deprived backgrounds impacted most significantly.^26^ It is imperative that, as we rebuild our society post‐pandemic, we address these growing disparities.

Promisingly, the Boards of NHS England and NHS Improvement acknowledge that the ‘pandemic has shone a stark light on inequalities in health and healthcare’,^27^ prompting the launch of the ‘Core20PLUS5’ initiative, which seeks to improve health access, experience and outcomes for the most deprived quintile of the population (as determined by the Index of Multiple Deprivation).^28^ In particular, opportunities for progress have been identified in five‐specific domains: maternity, severe mental illness, chronic respiratory disease, early cancer diagnosis and hypertension. It is hoped that the programme's guidance and resource allocation will empower local providers to address the bespoke post‐pandemic health needs of the communities they serve, while contributing towards these national goals.

## 5 WHY NOW IS AN OPPORTUNE TIME TO GROW AND PROMOTE LIFESTYLE MEDICINE

With a stretched health service, growing waiting lists and exacerbation of various disease risk factors, the demand for patient‐driven interventions is particularly pressing. The COVID‐19 aftermath presents a unique opportunity for cultivation of the Lifestyle Medicine field for several reasons.

Firstly, individuals, communities and businesses are likely to be particularly receptive to change. The media has fixated on epidemiological data implicating specific characteristics and behaviours (such as smoking or obesity) as risk factors for COVID‐19 morbidity. Promisingly, a Public Health England (PHE) survey revealed that 70% UK adults feel motivated by the pandemic to implement lifestyle changes, highlighting the post‐Covid era as a pivotal time to capitalise on people's engagement with their own health.^29^ Furthermore, we have grown accustomed to making sacrifices to ‘protect the NHS’. Since smoking, obesity, alcohol and physical inactivity cost the NHS £14 bn per year,^30^ one hopes that, with appropriate patient empowerment, we can continue to pull together as a nation to mitigate our burden on health services. Additionally, policy changes promoting lifestyle modifications could be integrated into plans to rebuild society. For example, workers transitioning back to the office could be supported to walk/cycle their commute or prepare nutritious lunches rather than buying processed ‘on‐the‐go’ food. Indeed, the government oversees many institutions responsible for feeding some of society's most vulnerable individuals (including schools, hospitals and prisons), offering opportunity for intervention and education. With individuals and industries already braced for change, it may be easier to adopt a healthier ‘new normal’.

However, before we can expect individuals to strive for healthier lifestyles, systemic changes are needed to make healthy choices more accessible. The pandemic set precedent for scientists and clinicians to collaborate with politicians to guide national policy more publicly than ever before. When reflecting upon our COVID‐19 response, we must assess the impact of government decisions on curbing viral transmission, as well as establishing chronic health habits. For example, when easing Lockdown One restrictions, pubs and restaurants re‐opened over a month before indoor gyms, pools and sports facilities. In addition to tempting individuals towards unhealthy behavioural patterns, such policy may have conveyed the message that maintaining physical and mental fitness is less ‘essential’ than consuming alcohol or eating out. Although the long‐term psycho‐social consequences are difficult to quantify, the importance of consulting Lifestyle Medicine advocates when implementing future policy is clear.

Whilst we have focussed on the detrimental impacts of COVID‐19, it is important to recognise that facets of Lifestyle Medicine became more accessible for certain populations. For example, 51% of UK survey respondents cooked more regularly during the pandemic (perhaps with closure of food‐outlets and more time at home). Promisingly, 82% expect to continue this habit.^31^ We must harness this renewed engagement with food preparation to reduce reliance on highly processed ready‐meals or takeaways.

Now is also an appropriate time to utilise the infrastructure developed in 2020 for Public Health England's ‘Better Health’ campaign. For example, the NHS Weight Loss Plan mobile app has already secured national awareness (864,403 downloads) and impressive efficacy amongst those who follow it to completion (averaging 5.8 kg weight loss over 12 weeks).^32^ However, further input from Lifestyle Medicine experts could enhance this service by addressing the high attrition rate (<1% of downloaders completed the programme^32^), tackling ethnic discrepancies in patient retention and integrating support for sleep, mental wellbeing and smoking/alcohol cessation for holistic health management.

## 6 CONCLUSION

COVID‐19 has exposed a global failure to adequately address the health consequences of many modern lifestyles. The pandemic has simultaneously promoted the importance of maintaining health‐protecting behaviours, while hampering many patients’ efforts to live healthily and exacerbating health inequalities. Although Lifestyle Medicine is inherently driven by personal needs/behaviours, individuals must be empowered by their environment and public policy to adopt healthy habits. Indeed, for us to truly ‘build back better’ and diminish our vulnerabilities to future threats, clinicians, scientists and politicians must collaborate to capitalise on these unique circumstances in order to construct a society, where the healthy choice becomes the easy choice.

## CONFLICT OF INTEREST

The authors declare that there is no conflict of interest that could be perceived as prejudicing the impartiality of the research reported.

## FUNDING INFORMATION

The authors received no specific funding for this work.

## Data Availability

Data sharing is not applicable to this article as no new data were created or analysed.
